# Optical imaging correlates with magnetic resonance imaging breast density and reveals composition changes during neoadjuvant chemotherapy

**DOI:** 10.1186/bcr3389

**Published:** 2013-02-22

**Authors:** Thomas D O'Sullivan, Anaïs Leproux, Jeon-Hor Chen, Shadfar Bahri, Alex Matlock, Darren Roblyer, Christine E McLaren, Wen-Pin Chen, Albert E Cerussi, Min-Ying Su, Bruce J Tromberg

**Affiliations:** 1 Laser Microbeam and Medical Program, Beckman Laser Institute and Medical Clinic, University of California, 1002 Health Sciences Road, Irvine, CA 92617, USA; 2 Tu & Yuen Center for Functional Onco-Imaging, Department of Radiological Sciences, University of California, 164 Irvine Hall, Irvine, CA 92617, USA; 3 Department of Radiology, E-Da Hospital and I-Shou University, 1 Yi-Da Road, Jiau-shu Tsuen, Yan-Chau Shiang, Kaohsiung 82445, Taiwan; 4 Current address: Department of Biomedical Engineering, Boston University, 44 Cummington Street, Boston, MA 02215, USA; 5 Chao Family Comprehensive Cancer Center, University of California, Irvine Medical Center, 101 The City Drive, Orange, CA 92868, USA; 6 Department of Epidemiology, University of California, 224 Irvine Hall, Irvine, CA 92697, USA

## Abstract

**Introduction:**

In addition to being a risk factor for breast cancer, breast density has been hypothesized to be a surrogate biomarker for predicting response to endocrine-based chemotherapies. The purpose of this study was to evaluate whether a noninvasive bedside scanner based on diffuse optical spectroscopic imaging (DOSI) provides quantitative metrics to measure and track changes in breast tissue composition and density. To access a broad range of densities in a limited patient population, we performed optical measurements on the contralateral normal breast of patients before and during neoadjuvant chemotherapy (NAC). In this work, DOSI parameters, including tissue hemoglobin, water, and lipid concentrations, were obtained and correlated with magnetic resonance imaging (MRI)-measured fibroglandular tissue density. We evaluated how DOSI could be used to assess breast density while gaining new insight into the impact of chemotherapy on breast tissue.

**Methods:**

This was a retrospective study of 28 volunteers undergoing NAC treatment for breast cancer. Both 3.0-T MRI and broadband DOSI (650 to 1,000 nm) were obtained from the contralateral normal breast before and during NAC. Longitudinal DOSI measurements were used to calculate breast tissue concentrations of oxygenated and deoxygenated hemoglobin, water, and lipid. These values were compared with MRI-measured fibroglandular density before and during therapy.

**Results:**

Water (*r *= 0.843; *P *< 0.001), deoxyhemoglobin (*r *= 0.785; *P *= 0.003), and lipid (*r *= -0.707; *P *= 0.010) concentration measured with DOSI correlated strongly with MRI-measured density before therapy. Mean DOSI parameters differed significantly between pre- and postmenopausal subjects at baseline (water, *P *< 0.001; deoxyhemoglobin, *P *= 0.024; lipid, *P *= 0.006). During NAC treatment measured at about 90 days, significant reductions were observed in oxyhemoglobin for pre- (-20.0%; 95% confidence interval (CI), -32.7 to -7.4) and postmenopausal subjects (-20.1%; 95% CI, -31.4 to -8.8), and water concentration for premenopausal subjects (-11.9%; 95% CI, -17.1 to -6.7) compared with baseline. Lipid increased slightly in premenopausal subjects (3.8%; 95% CI, 1.1 to 6.5), and water increased slightly in postmenopausal subjects (4.4%; 95% CI, 0.1 to 8.6). Percentage change in water at the end of therapy compared with baseline correlated strongly with percentage change in MRI-measured density (*r *= 0.864; *P *= 0.012).

**Conclusions:**

DOSI functional measurements correlate with MRI fibroglandular density, both before therapy and during NAC. Although from a limited patient dataset, these results suggest that DOSI may provide new functional indices of density based on hemoglobin and water that could be used at the bedside to assess response to therapy and evaluate disease risk.

## Introduction

Breast density, an assessment of the volume fraction of the human breast that contains epithelial and connective tissues, is a risk factor for breast cancer. Numerous studies have shown that women with the highest density category evaluated from mammography have fourfold to sixfold increased cancer risk compared with women with lower density [1]. In addition, the International Breast Cancer Intervention Study (IBIS-I) primary chemoprevention study, evaluating the selective estrogen-receptor modulator (SERM) tamoxifen [2,3], revealed that only women who exhibited >10% reduction in percentage mammographic density experienced the protective effect of tamoxifen in decreased cancer incidence [4]. Women in the treated group who showed <10% decreased density had exactly the same cancer rates compared with the control group. Similar results were also recently found regarding the use of tamoxifen and aromatase inhibitors in the adjuvant setting [5]. These and related studies suggest that breast tissue density, in addition to being a risk factor, may also be a surrogate biomarker for monitoring, predicting, and optimizing individual response to hormonal therapies. However, methods to measure breast density by mammography or MRI have not been adopted for various reasons, preventing the utilization of breast-density measurements in the clinic to assess risk or predict outcome.

The only established criterion for assessing breast density is given by the Breast Imaging Reporting and Data System (BI-RADS) [6]. That system defines four categories for breast density, qualitatively based on the relative amounts of fat and dense fibroglandular tissue in a mammogram. Although the system is useful for evaluating the probability that a tumor is obscured by dense tissue on a mammogram, it is not suitable for quantifying or measuring small changes in density. An alternative approach is to quantify PMD by using computer-based image-analysis techniques of mammograms [7-11]. Whereas it is possible to quantify density from the image, a 2D radiograph projection is inherently limited in its ability to quantify longitudinal density changes accurately [12]. The use of ionizing radiation also limits the frequency of measurements, making it unsuitable for monitoring adjuvant or preventive chemotherapy. We and others have investigated the use of MRI [12-19], which is a safe and quantitative technique for measuring breast density and volume, but its high cost precludes it from being applied for risk assessment and screening in most women.

Optical-based imaging modalities are a promising alternative to characterize breast density. Because of the absorption and scattering properties of breast tissue, near-infrared light (650 to 1,000 nm) is able to penetrate several centimeters deep. Researchers have measured breast tissue in transmission and reflectance geometries by using continuous-wave spectroscopy, frequency-domain, or time-domain techniques [20,21]. To correlate tissue optical measurements with breast density, several groups have compared spectroscopic features or measured tissue components (such as blood, water, and lipid) with mammographic density. These optically measured parameters have been compared against breast densities that have been qualitatively analyzed from mammograms, classified into only three or four density categories [22-28]. As mentioned earlier, this limits the ability of the measurement to detect small density changes. Promising work attempted to correlate mammographic density with transillumination optical spectroscopy [29,30], but these studies did not quantify tissue scattering and biochemical composition. This makes it difficult to compare patient spectroscopic features and to link functional changes with underlying mechanisms of breast density.

We hypothesize that diffuse optical spectroscopic imaging (DOSI) provides quantitative metrics to measure and track changes in breast tissue composition and density. DOSI provides a quantitative measure of tissue functional components, allowing noninvasive imaging of breast tissue composition and metabolism [31]. DOSI is capable of measuring tissue concentrations (ct) of oxygenated hemoglobin (ctO_2_Hb), deoxygenated hemoglobin (ctHHb), water, and lipid. These measurements are directly related to tissue metabolism and vascular characteristics. For example, high levels of ctO_2_Hb are considered to be a surrogate marker for elevated vascular supply and perfusion. High levels of ctHHb reflect high oxygen consumption and tissue metabolism due to cell proliferation and/or poor vascular drainage. Total hemoglobin (ctTHb) corresponds to the total blood volume in tissue and has been validated as an index that corresponds to increased vascular density [32].

DOSI scanning is performed without compression or injection of contrast agents by using a bedside hand-held probe. Much of this work has been focused on determining functional changes in breast tumors during chemotherapy, a topic that is now under investigation in an American College of Radiology Imaging Networks (ACRIN) multicenter clinical trial [33]. The DOSI technique combines laser-based frequency-domain photon migration with broadband near-infrared spectroscopy to separate optical absorption and scattering over a broad spectral range [20]. This results in a quantitative dataset of tissue concentrations that can be compared longitudinally in the same patient, or across different patients.

Our previous studies [34-36] showed that premenopausal women tend to have greater water concentration than do postmenopausal women, reflecting the high water content of epithelial connective-tissue compartments. Similarly, premenopausal women have a higher hemoglobin concentration (both ctHHb and ctO_2_Hb), because of greater vascular demands of the glandular tissue, and a lower lipid concentration. Based on these data, we expect that breast density, which quantifies the abundance of hormonally controlled glandular tissue, exhibits a positive correlation with water and ctTHb and a negative correlation with lipid.

Because it is known that neoadjuvant chemotherapy (NAC) affects the density and composition of normal breast [37], in this study, we measured the contralateral normal side of breast cancer patients before and during NAC treatment with MRI and DOSI. We examined baseline composition, comparing differences between pre- and postmenopausal women. We analyzed DOSI parameters for markers of breast density and metabolism. The correlation between these results and fibroglandular tissue density measured with MRI was examined to test the hypothesis that DOSI can provide a quantitative measure of breast density. We further hypothesize that, in addition to providing an optical index of breast density, DOSI may help provide insight into mechanisms of chemotherapy-induced changes in breast metabolism.

## Methods

### Subject measurements

This study is a retrospective analysis conducted on a subset of subjects with newly diagnosed, operative, primary breast cancer measured with DOSI or DOSI+MRI during their neoadjuvant chemotherapy treatment between 2007 and 2012. Subject demographics are shown in Table 1. DOSI measurements were acquired at a minimum of 30 locations (taken in a rectangular grid pattern with 10-mm spacing between measurement points) on a contralateral breast not suggestive of malignancies at two or more time points during the first 120 days of therapy (*n *= 28). The DOSI+MRI cohort is a subset (*n *= 12) of subjects who also received MRI imaging before NAC. Post-NAC MRI images were available for nine subjects.

**Table 1:** Subject demographics

	Age (years) mean ± SD	Number
DOSI cohort	47.4 ± 10.2	28
Premenopausal	40.8 ± 5.0	17
Postmenopausal	57.6 ± 7.2	11
DOSI+MRI cohort	43.6 ± 11.0	12
Premenopausal	38.l ± 5.3	9
Postmenopausal	60.0 ± 4.0	3

DOSI, diffuse optical spectroscopic imaging.

All subjects provided informed written consent and participated in this study under clinical protocols approved by the Institutional Review Board at the University of California, Irvine (2002-2306, 2007-6084, and 2011-7812). Exclusion criteria included pregnant women and women who were younger than 21 years or older than 75 years. All subjects were histologically diagnosed with invasive carcinoma before neoadjuvant treatment.

### DOSI measurement

A comprehensive description of the diffuse optical spectroscopic imaging (DOSI) system and underlying concepts may be found elsewhere [38-40]. In brief, the instrument combines frequency domain photon migration (FDPM) and continuous-wave near-infrared spectroscopy (CW-NIRS) measurements to determine the optical scattering and absorption spectra (650 to 1,000 nm) of the measured tissue. The FDPM component consists of six laser diode sources (660, 680, 780, 810, 830, and 850 nm) that are sinusoidally intensity modulated between 50 and 500 MHz. The relative amplitude and phase of the detected signals compared with the source are input into an analytic model of diffuse light transport to determine tissue scattering and absorption coefficients at these wavelengths. White-light illumination at each measurement point is used for CW-NIRS spectroscopy. The detected broadband reflectance spectra are fit and scaled to the frequency-domain scattering and absorption measurements to obtain full broadband absorption spectra over the entire spectral range. Absolute tissue concentrations are calculated by using the Beer-Lambert law and known extinction coefficient spectra of ctHHb, ctO_2_Hb, water, and bulk lipid.

All subjects received NAC before surgical resection of tumors and were measured with the DOSI system before treatment (to establish a baseline measurement), and at several time points throughout their treatment. Based on our previous findings, baseline measurements were obtained at least 10 days after diagnostic biopsies to minimize their impact on DOSI scans [41]. Subjects were measured in a supine position. The DOSI probe was placed against the breast tissue, and sequential measurements were taken in a linear or rectangular grid pattern by using 10-mm spacing. Measurement regions on the normal breast were taken to mirror the area of the underlying tumor determined by ultrasound and palpation on the ipsilateral breast. Total measurement time varied between 20 minutes and 1 hour per subject. Repeated DOSI scans were shown previously to be relatively insensitive to probe-contact pressure fluctuations, displaying less than 5% average variation in test/retest studies of human subjects [42].

### Mammographic density analysis

Each subject was characterized by a BI-RADS density category. In this system, category I is described as fatty breast tissue, II is scattered density, III is heterogeneously dense, and IV is extremely dense. The categories were compiled from prechemotherapy mammographic reports documented by the subjects' radiologists and were available for 20 of the 28 subjects. Of these, four subjects were BI-RADS density II, eleven subjects were BI-RADS III, and five subjects were BI-RADS IV.

### MR imaging and breast-density analysis

MR imaging examinations were performed by using a dedicated sensitivity-encoding-enabled bilateral four-channel breast coil with a 3.0-T system (Achieva; Philips Medical Systems, Best, The Netherlands) at time points before, during, and after completion of NAC. The axial-view T_1_-weighted images without fat suppression were used for the analysis of breast density in this study. The images were acquired by using a 2D turbo spin-echo pulse sequence with TR, 800 milliseconds; TE, 8.6 milliseconds; flip angle, 90 degrees; matrix size, 480 × 480; FOV, 31 to 38 cm; and slice thickness, 2 mm.

The breast and fibroglandular tissue segmentation was performed by using a modified published method [12,43,44]. Before the segmentation, the operator viewed the whole axial T_1_W images dataset and determined the superior and inferior boundaries of the breast (the beginning and ending slices) by comparing the thickness of breast fat with that of the body fat. The breast-segmentation procedures consisted of (a) an initial horizontal line cut along the posterior margin of each individual subject's sternum to exclude thoracic region; (b) Applied Fuzzy-C-Means (FCM) clustering and b-spline curve fitting to obtain the breast-chest boundary; (c) a bias field-correction method based on nonparametric nonuniformity normalization (N3), and an adaptive FCM algorithm [44] was used to remove the strong intensity non-uniformity for segmentation of fibroglandular tissue and fatty tissue; (d) applied dynamic searching to exclude the skin along the breast boundary; and (e) the standard FCM algorithm was applied to classify all pixels on the image. The default setting was to use a total of six clusters, three for fibroglandular tissue and three for fatty tissues. After completing the segmentation processes in all image slices, the quantitative breast volume, fibroglandular tissue volume, and the percentage density (calculated as the ratio of the fibroglandular tissue volume to the breast volume ×100%) were calculated.

### Neoadjuvant chemotherapy regimen

Most patients (*n *= 19) received a 12-cycle, once-a-week course of a paclitaxel (either albumin-bound (nab-paclitaxel) or Cremophor-bound) and carboplatin. Other patients received only doxorubicin+cyclophosphamiden (AC therapy) (*n *= 2), or received additional AC therapy either before (*n *= 4) or after (*n *= 2) paclitaxel+carboplatin. The remaining patient received docetaxel+carboplatin for six cycles, once every 3 weeks. Many patients also received bevacizumab as part of their treatment (*n *= 15), and some HER2/neu-positive patients also received trastuzumab (*n *= 8).

### Statistical analysis

#### Data description

Summary statistics including mean and standard error were calculated for the DOSI-measured parameters of water, bulk lipid, ctO_2_Hb, ctHHb, ctTHb, oxygen saturation (stO_2_), and tumor optical index (TOI) measured at baseline and during NAC. TOI is a tissue optical index of metabolism that provides contrast for metabolically active tissue, developed for identifying tumors [45], and is given by TOI = ctHHb × water/(% lipid). Subject measurements during NAC were recorded as having occurred in one of four intervals with interval midpoints 30 days (mean, 33.1; range, 21 to 43/*n *= 16 pre-, *n *= 11 postmenopausal), 60 days (mean, 60.8; range, 55 to 69/*n *= 10 pre-, *n *= 7 postmenopausal), 90 days (mean, 89.9; range, 78 to 104/*n *= 16 pre-, *n *= 9 postmenopausal), and 120 days (mean, 116.6; range, 106 to 127/*n *= 6 pre-, *n *= 4 postmenopausal) from the beginning of chemotherapy treatment.

#### Comparison of DOSI parameters between groups

The Mann-Whitney *U *test was applied to test whether mean values for DOSI parameters differed significantly between pre- and postmenopausal subjects. The Shapiro-Wilkes test was applied to each DOSI parameter to test the fit to a normal distribution within BI-RADS categories II, III, and IV. Analysis of variance (ANOVA) was applied to compare mean values among BI-RADS categories for water, lipid concentration, ctO_2_Hb, ctHHb, ctTHb, and scattering power. The nonparametric Kruskal-Wallis test was applied to compare the distributions of stO_2 _and TOI values among BI-RADS categories. For pairs of BI-RADS categories (II versus III, II versus IV, and III versus IV), the mean values for DOSI parameters were compared with application of the Bonferroni-Holm method of adjustment for multiple comparisons to maintain an experiment-wise significance level of 0.05 for each DOSI parameter.

#### Regression and correlation analyses

The linear relation between age and water concentration measured at baseline was assessed with linear regression analysis and the Pearson correlation coefficient. The influence of values obtained from individual patients was assessed by examination of regression residuals and the DFFITS statistic. In addition, the correlation between age and change in water concentration at 90 days during NAC was estimated with the Pearson correlation coefficient.

The correlation between DOSI parameters and MRI fibroglandular density measured at baseline, as well as at 24 days and 82 days during NAC, was assessed with the Pearson correlation coefficient. Similarly, the correlation between percentage change in DOSI parameters and percentage change in MRI fibroglandular density at 82 days of NAC (that is, the end of NAC treatment) was assessed with the Pearson correlation coefficient. A significance level of 0.05 was used for assessment of estimated correlations.

#### Generalized estimating equations

We applied a statistical method known as generalized estimating equations (GEEs) to estimate and compare the expected (mean) change from baseline for each specified DOSI parameter between pre- and postmenopausal subjects. For example, the GEE method was used to model the linear relation between the mean change in breast-tissue water as a function of predictors including menopausal status, measurement day after NAC, and the interaction between menopausal status and measurement day. Measurement day from the beginning of chemotherapy was represented by a categorical variable with four categories. In contrast to ordinary linear regression, for which values measured in individual subjects are assumed to be independent, the GEE method takes in account the correlation between DOSI values measured within individual subjects. For application of the GEE, it is necessary to specify nature of the linear relation between the mean value of the DOSI parameters and the predictors and between the mean and variance of DOSI parameter values. The nature of the within-subject correlations between DOSI measurements must also be specified. In technical language, we specified a normal model with an identity-link function and an exchangeable correlation structure. In brief, these specifications indicated that the mean and variance of the DOSI parameter are related through a normal distribution and that the within-subject correlation between repeated measurements of DOSI values was assumed to be the same for each subject.

From the final GEE model for a given outcome, the estimated percentage change from baseline was calculated and compared for both the pre- and the postmenopausal groups 30, 60, 90, and 120 days from the beginning of chemotherapy. For analysis of each outcome, the Bonferroni-Holm method of adjustment for multiple comparisons was applied to maintain an experiment-wise significance level of 0.05.

## Results

**Figure 1:**
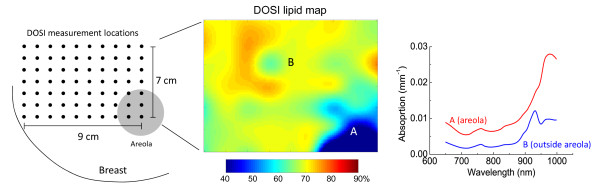
**Diffuse optical spectroscopic imaging (DOSI) measurement schematic**. A two-dimensional map of functional properties is generated by measuring broadband absorption and scattering spectra in a grid pattern. On the left is a grid (10 mm) in which each dot represents a DOSI measurement point. The middle is a map of lipid concentration (actual breast data) generated from the measured spectra on the right. Note that the areola region **(A) **has much less lipid concentration and overall higher optical absorption compared with the rest of the breast **(B) **because of the higher concentration of water and hemoglobin in fibroglandular tissue.

For each patient examination, tissue concentrations of ctO_2_Hb, ctHHb, water, and lipid were calculated at each measurement point from the broadband absorption spectra. These data were used to construct 2D maps by using a linear interpolation between measurement points, as shown in Figure 1. The areolar region provides significant contrast because of the high density of fibroglandular tissue and its increased metabolic activity. Optical absorption spectra of tissue in this region show higher concentrations of hemoglobin and reduced lipid content. For analysis, the average of DOSI measurement parameters (chromophore concentrations and scattering coefficients) was computed over the entire measurement region but excluding the areola. Because the areolar region is a concentrated region of fibroglandular tissue not representative of the breast as a whole, it was excluded from the DOSI average for this analysis; the nipple also was excluded from MRI segmentation of fibroglandular tissue. Identical measurement grids were used for longitudinal analysis.

### Baseline pre- and postmenopausal differences

**Figure 2:**
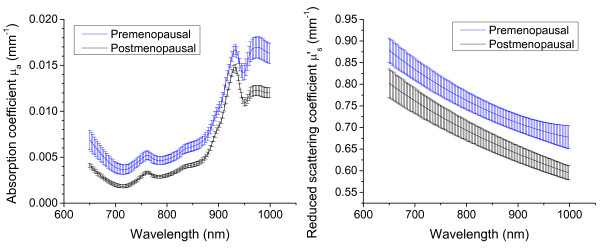
**Average absorption and scattering spectra measured at baseline for premenopausal (*n *= 17) and postmenopausal (*n *= 11) subjects**. First, the average value was computed for measurements within each subject, and then the means for the resulting spectra of all subjects were computed. Error bars represent standard error.

Figure 2 shows the average optical absorption and scattering spectra over all measurement points, excluding the areola, for all premenopausal (*n *= 17) and postmenopausal (*n *= 11) subjects. Discernible differences are present in both the absorption and scattering spectra between premenopausal and postmenopausal women. Premenopausal women exhibit higher concentrations of hemoglobin, as evidenced by the overall higher absorption in the 670- to 850-nm range. Increased tissue water concentration exists relative to lipids in premenopausal women, as revealed by the large water-absorption peak at 980 nm compared with the lipid peak at 930 nm.

Table 2 shows the absolute DOSI parameters for premenopausal, postmenopausal, and all subjects at baseline before beginning NAC treatment. In the absence of chemotherapeutic intervention, pre- and postmenopausal subjects exhibited statistically significant difference in means for water (*P *< 0.001), lipid (*P *= 0.006), ctHHb (*P *= 0.024), and the tissue optical index (TOI) (*P *= 0.003). Postmenopausal women had a lower mean ctHHb and mean water concentration at baseline than did premenopausal women, and a higher mean lipid concentration.

**Table 2:** Absolute tissue concentrations (mean ± standard error) measured in the normal breast at baseline

	Water (%)	Lipid (%)	ctO_2_Hb (µ*M*)	ctHHb (µ*M*)	ctTHb (µ*M*)	stO_2 _(%)	TOI
All *n *= 28	21.4 ± 1.3	69.7 ± 1.3	18.0 ± 1.2	5.0 ± 0.2	23.0 ± 1.3	77.2 ± 0.9	1.7 ± 0.2
Pre *n *= 17	24.4 ± 1.8^c^	67.0 ± 1.6^b^	18.9 ± 1.7	5.3 ± 0.2^a^	24.2 ± 1.9	76.8 ± 1.1	2.1 ± 0.3^b^
Post *n *= 11	16.6 ± 0.7^c^	74.0 ± 1.4^b^	16.6 ± 1.5	4.5 ± 0.2^a^	21.1 ± 1.6	77.7 ± 1.7	1.0 ± 0.1^b^

Data are shown for all patients, and for the premenopausal (pre) and postmenopausal (post) subgroups. ctO_2_Hb, oxyhemoglobin concentration; ctHHb, deoxyhemoglobin concentration; ctTHb, total hemoglobin concentration; stO_2_, tissue oxygen saturation; TOI, tissue optical index. ^a,b,c^A statistically significant difference (Mann-Whitney *U *test) between pre- and postmenopausal groups (*P *< 0.05; *P *< 0.01; *P *< 0.001, respectively).

**Figure 3:**
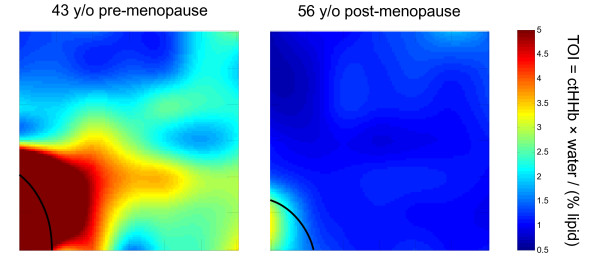
**Typical maps of tissue optical index (TOI) in the breast of a premenopausal and postmenopausal subject at baseline**. The outer limit of the areolar region is indicated by the black line. Tick-mark separation equals 1 cm. ctHHb, deoxyhemoglobin concentration.

Figure 3 shows maps of TOI for a typical premenopausal and a postmenopausal subject. In both subjects, the areolar region (indicated by the black line) exhibits much higher TOI than does the surrounding tissue, whereas the surrounding TOI tends to be higher in the premenopausal subjects.

**Figure 4:**
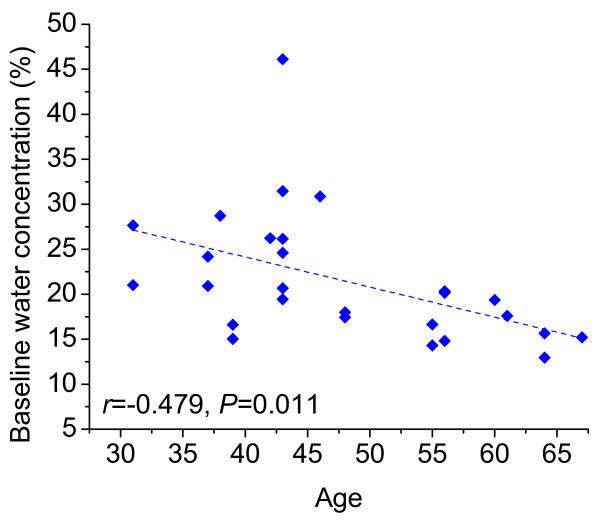
**Breast-tissue water concentration at baseline decreased with age (*r *= -0**.479; *P *= 0.011). Data shown are for 27 pre- and postmenopausal subjects (one patient was excluded because she previously underwent an oophorectomy, confounding the effect of hormones on breast density).

Because the mean tissue water concentration in the normal breast was significantly different at baseline between pre- and postmenopausal groups, the relation between age and water concentration was examined to explore these differences in more detail. For a subgroup of 27 subjects (one patient was excluded as she previously underwent an oophorectomy, which caused premature menopause and confounds the effect of hormones on breast density), water concentration at baseline exhibited significant negative correlation (*r *= -0.479; *P *= 0.011) with age (Figure 4). One subject with extremely high breast density and corresponding water concentration (46.1%) exerted substantial influence on the regression coefficients, as indicated by regression diagnostics. Previous studies by our group and others have shown that water concentration can vary dramatically in premenopausal patients [36,46,47], perhaps because of normal fluctuations caused by the menstrual cycle [48], which may account for the outlier.

### Relation between DOSI parameters and mammographic density categories

**Figure 5:**
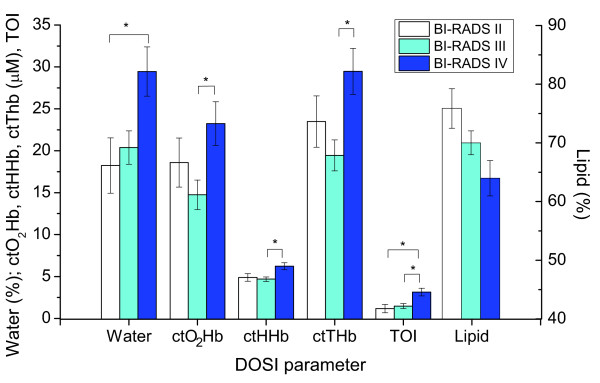
**The mean value of measured diffuse optical spectroscopic imaging parameters, separated by the Breast Imaging-Reporting and Data System (BI-RADS) density category**. A statistically significant difference (*) was found between BI-RADS density categories III and IV for oxyhemoglobin (ctO_2_Hb), deoxyhemoglobin (ctHHb), total hemoglobin (ctTHb), and tissue optical index (TOI). Additionally, a statistically significant difference was found between BI-RADS II and IV for water and TOI. Error bars represent standard error.

The relations between DOSI parameters and mammographic-density categories, based on the four traditional BI-RADS density categories, were assessed (Figure 5; all data shown in Additional file 1, Table S1.). None of the subjects measured was characterized as BI-RADS I. A statistically significant difference was found between BI-RADS density categories III and IV for ctO_2_Hb, ctHHb, ctTHb, and TOI. Additionally, a statistically significant difference was found between BI-RADS II and IV for water and TOI. Mean lipid concentration tended to decrease with increasing BI-RADS density category and approached statistical significance. No significant difference in the means of stO_2 _or scattering power was found.

### Correlation between DOSI parameters and MRI breast density

**Figure 6:**
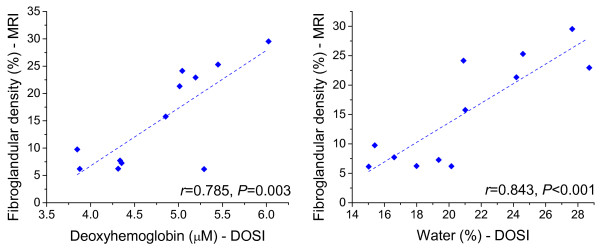
**Correlation between optically measured deoxyhemoglobin (ctHHb) and water, with fibroglandular density measured with magnetic resonance imaging (MRI)**. Data are for 12 subjects (all subjects for whom we had available corresponding data) at baseline before chemotherapy (nine pre- and three postmenopausal). The fitted linear regression line is superimposed on the graph.

**Figure 7:**
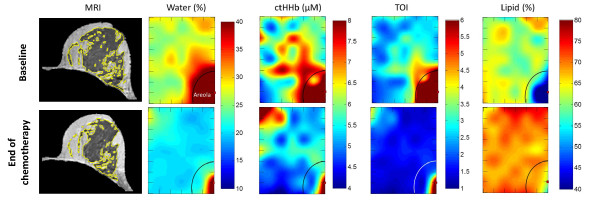
**Corresponding magnetic resonance imaging (MRI) and diffuse optical spectroscopic imaging (DOSI)**. Images were taken at baseline and end of neoadjuvant chemotherapy in the contralateral normal breast of a premenopausal patient. The yellow outlines in the MRI images depict the result of the segmentation algorithm for fibroglandular tissue in the shown slices. The DOSI maps depict measured parameters as a function of position (tick mark separation equals 1 cm). The illustrated maps are from an 8- × 6-cm area from the upper-inner region of the left breast. The areolar region has more water, outlined by the semicircle. The decreased density after chemotherapy is clearly visible in both MRI and DOSI. MRI shows 30.4% reduction in fibroglandular tissue volume, and DOSI shows 24.4% reduction in tissue water. ctHHb, deoxyhemoglobin concentration; TOI, tissue optical index.

The correlations between measured DOSI parameters and MRI fibroglandular tissue volume were examined at baseline and at various time points during NAC (Table 3). At baseline, breast density calculated from MRI showed stronger correlations with ctHHb (*r *= 0.785; *P *= 0.003), water concentration (*r *= 0.843; *P *< 0.001), lipid (*r *= -0.707; *P *= 0.010), and TOI (*r *= 0.891; *P *< 0.001) than with other measures. Figure 6 illustrates the linear relation between water and ctHHb with breast density. A statistically significant correlation with ctTHb (*r *= 0.597; *P *= 0.040) was also demonstrated. Figure 7 displays the MR images and DOSI images at the beginning and end of NAC for a 31-year-old premenopausal subject. This subject exhibited a significant reduction in breast density during NAC, and, similarly, a significant reduction in water concentration. However, when correlations between MRI breast density and DOSI parameters were estimated at time points near the conclusion of NAC treatment (that is, about 82 days), nonsignificant correlations were found (*P *> 0.05 for all DOSI parameters).

**Table 3:** Correlation between diffuse optical spectroscopic imaging parameters and fibroglandular density measured with magnetic resonance imaging for subjects at baseline and at time points during chemotherapy

	Baseline (*n *= 12)		~25 days (*n *= 10) Mean, 24.8 ± 5.5		~82 days (*n *= 7) Mean, 82.4 ± 11.9	
	
	*R*	*P*	*R*	*P*	*r*	*P*
Water	**0.843**	**<0.001**	**0.675**	**0.032**	0.405	0.367
Lipid	**-0.707**	**0.010**	-0.462	0.179	-0.018	0.970
ctO_2_Hb	0.557	0.060	**0.647**	**0.043**	0.464	0.294
ctHHb	**0.785**	**0.003**	0.399	0.253	0.705	0.077
ctTHb	**0.597**	**0.040**	**0.644**	**0.045**	0.599	0.155
stO_2_	0.196	0.543	0.240	0.505	0.012	0.979
TOI	**0.891**	**<0.001**	0.541	0.107	0.593	0.160
Scat. power	0.205	0.523	0.143	0.694	0.265	0.566

Bold values indicate statistical significance at the 0.05 level. ctO_2_Hb, oxyhemoglobin concentration; ctHHb, deoxyhemoglobin concentration; ctTHb, total hemoglobin concentration; stO_2_, tissue oxygen saturation; TOI, tissue optical index; Scat. power, optical scattering power.

We also examined the correlation between percentage change compared with baseline of the optical parameters and MRI fibroglandular density at about 82 days (Table 4). A strong correlation of both change in water (*r *= 0.864; *P *= 0.012) and TOI (*r *= 0.818; *P *= 0.025) with MRI was found.

**Table 4:** Correlation between percentage change in optical parameters and percentage change in fibroglandular density by magnetic resonance imaging

	*r*	*P*
Water	**0.864**	**0.012**
Lipid	-0.502	0.251
ctO_2_Hb	-0.166	0.721
ctHHb	0.606	0.149
ctTHb	0.017	0.971
stO_2_	-0.412	0.357
TOI	**0.818**	**0.025**
Scat. power	0.195	0.675

Data are for the seven subjects measured at approximately 82 days of chemotherapy (five pre- and two postmenopausal). Bold values indicate statistical significance at the 0.05 significance level. ctO_2_Hb, oxyhemoglobin concentration; ctHHb, deoxyhemoglobin concentration; ctTHb, total hemoglobin concentration; stO_2_, tissue oxygen saturation; TOI, tissue optical index; Scat. power, optical scattering power.

### Variations in breast composition during NAC

**Figure 8:**
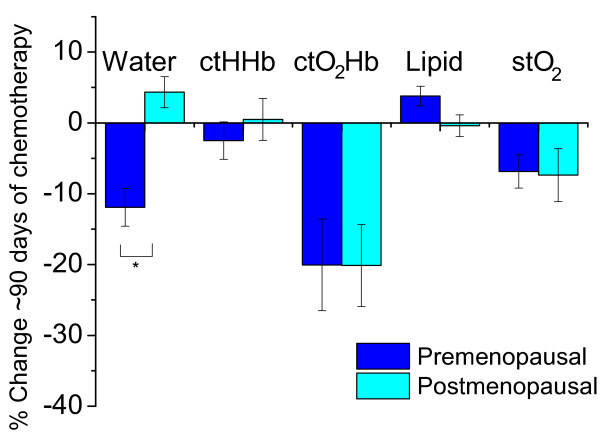
**Percentage change in water, deoxyhemoglobin (ctHHb), oxyhemoglobin (ctO_2_Hb), lipid, and tissue oxygen saturation (stO_2_) at 90 days of chemotherapy treatment compared with baseline**. Based on longitudinal generalized estimating equation models, a statistically significant difference in water (*) was found between premenopausal and postmenopausal groups. Error bars represent standard error.

Significant compositional changes of the normal breast were observed during NAC in both pre- and postmenopausal subjects with DOSI. GEE models that incorporated menopausal status and measurement day were used to fit the outcomes of percentage change of DOSI parameter from baseline. Because most NAC regimens lasted 12 weeks, we show the GEE results at about 90 days from the beginning of NAC treatment (Figure 8). The measured DOSI parameters at baseline before NAC and at approximately 30, 60, 90, and 120 days after the start of NAC are shown in Additional file 2, Table S1. Both the premenopausal (-20.0%; 95% CI, -32.7 to -7.4) and postmenopausal (-20.1%; 95% CI, -31.4 to -8.8) groups exhibited statistically significant decreases in ctO_2_Hb, whereas ctHHb stayed relatively flat (premenopausal, -2.5%; 95% CI, -7.7 to 2.7; postmenopausal, 0.5%; 95% CI, -5.3 to 6.3), yielding a reduction of ctTHb. Bulk lipids also remained relatively flat during NAC for both pre- (3.8%; 95% CI, 1.1 to 6.5) and postmenopausal (-0.4%; 95% CI, -3.4 to 2.6) groups. The stO_2 _was nearly identical between the two groups at baseline, and both decreased in a similar manner during NAC (premenopausal, -6.9%; 95% CI, -11.5 to -2.3; postmenopausal, -7.4%; 95% CI, -14.7 to 0.0). Scattering amplitude and power did not change appreciably during NAC or between groups (data not shown).

**Figure 9:**
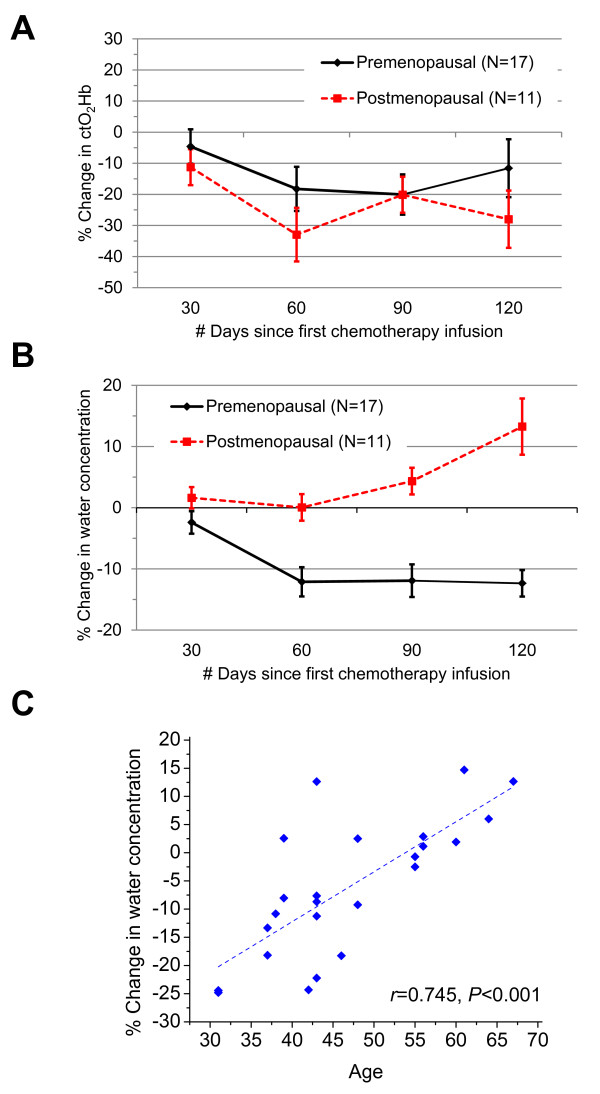
**Optically measured changes over time during chemotherapy**. **(A) **Oxyhemoglobin concentration (ctO_2_Hb) decreased during chemotherapy (90 days) for both groups of patients. **(B) **Water concentration decreased in premenopausal patients during chemotherapy and remained relatively unchanged in postmenopausal patients. **(C) **The reduction of water observed after 90 days of chemotherapy was associated with age (*r *= 0.745; *P *< 0.001), with younger subjects exhibiting a greater reduction. Data shown are for 24 pre- and postmenopausal subjects in whom we had measurement data at 90 days (one patient was excluded because she previously underwent an oophorectomy, confounding the effect of hormones on breast density). The fitted linear regression line is superimposed on the graph.

Trends during NAC were similar in both menopause groups for all DOSI parameters except water (ctO_2_Hb, shown in Figure 9A), which exhibited a percentage change that was statistically different between menopause groups at 90 days. Figure 9B shows that the premenopausal group incurred a steady decrease in tissue water during NAC, whereas breast-tissue water concentration in the postmenopausal group remained flat. After 90 days of NAC, premenopausal subjects exhibited an estimated -11.9% (95% CI, -17.1 to -6.7) reduction in breast-tissue water concentration, whereas postmenopausal subjects showed an estimated increase of 4.4% (95% CI, 0.1 to 8.6). The percentage change in water after 90 days of chemotherapy was correlated with age (Figure 9C; *r *= 0.745; *P *< 0.001), providing further evidence that the effect of NAC on water concentration is stronger in younger, premenopausal subjects.

## Discussion

Breast density is a strong independent risk factor for breast cancer and may also have a role in the risk of recurrence. Optical imaging is a low-cost imaging modality that shows promise for assessing breast density in the clinic without the use of ionizing radiation. Because optical imaging measures functional characteristics of tissue, it also yields additional information that complements breast-density-assessment techniques using MRI and mammography, which are based primarily on structure. This additional information may help to elucidate the origin of breast density and to clarify causes of both natural and treatment-induced changes of breast density. By understanding the factors that modulate breast density and the source of its contrast in imaging, we will be able to apply the parameter better in risk assessment.

Conflicting information exists in the literature correlating optically measured tissue components with breast density. Most agree that increasing density correlates with increasing water concentration and ctTHb. However, conflicting data are found on other measures, including bulk lipid, which has found to be negatively correlated with density [27], or not at all [24]. Oxygen saturation (the fraction of ctO_2_Hb to ctTHb, or stO_2_) has been shown to be reduced in dense tissue [25], whereas others do not find a significant correlation [24,27]. In addition, Taroni *et al*. [27] included collagen as a basis chromophore and found that it is strongly associated with BI-RADS categories II-IV. Optical scattering is also expected to increase with greater breast density because of the underlying fibroglandular structures, and some studies have indeed shown this [25,27]. It is possible that these disagreements are due to the broad classification of density categories, to the limited ability of the applied optical modalities to quantify tissue components accurately, or to some combination of the two.

In this study, we found that water, ctO_2_Hb, ctHHb, ctTHb, and TOI are associated with the BI-RADS density category, whereas lipid tended to decrease with increasing category but was not statistically significant. The largest mean differences in hemoglobin were found between BI-RADS density categories III and IV, but not category II and IV. This contradiction is likely due to the small sample size (*n *= 4 with BI-RADS II) and the limitations of a broad density-classification scheme. We expect lipid to be significantly associated with the BI-RADS category in a larger sample size. No association was discovered between stO_2 _or scattering power with BI-RADS density.

Several DOSI parameters were strongly correlated with MRI fibroglandular breast density measured at baseline, including tissue water, lipid, and ctHHb concentrations. No significant correlation was noted between density and scattering parameters or stO_2_. This significant correlation with water is likely because fibroglandular tissue has about 30% higher water content than does adipose tissue [24]. These observations are in general agreement with the findings of other groups, with the addition that here we show, for the first time, that a quantitative measure of water can be compared longitudinally and between patients. Because DOSI is a measurement of chromophore concentration throughout the probed tissue volume, the increased water may also be due to the increased vascular supply required by the dense tissue. Furthermore, we demonstrated a strong baseline correlation between ctHHb and breast density, reflecting the increased rate of metabolism in fibroglandular breast tissue. Because metabolism is strongly captured in the tissue optical index (TOI), this metric was also shown to be a good predictor of breast density. Finally, greater hemoglobin concentrations were observed in patients with dense breasts, suggesting greater vascular density in these subjects, and lower lipid concentrations, as would be expected by the greater volume fraction of fibroglandular tissue compared to adipose.

At baseline, we observed statistically significant differences in the breast composition and indicators of metabolism for premenopausal and postmenopausal groups, confirming previous studies [47]. Increased mean ctHHb and mean water concentrations were found, as well as decreased mean bulk lipid concentration in the breasts of premenopausal subjects, compared with those of postmenopausal subjects. This is all consistent with increased cell proliferation and metabolic activity in the denser breast tissue of younger premenopausal women.

Significant changes in optical markers for vascular density and supply (ctO_2_Hb and water) were observed during NAC treatment. A significant decrease in ctO_2_Hb was observed in both premenopausal and postmenopausal groups. The steady reduction of ctO_2_Hb without a corresponding decrease in ctHHb suggests that NAC agents act directly on the breast tissue, perhaps by causing a reduction of perfusion. This may be caused by chemotherapy-induced vascular damage and may contribute to the reduction of breast density. In contrast, only premenopausal subjects experienced a significant loss of water in their breast tissue during NAC. This trend better matches the observed effect of NAC on MRI-measured breast density wherein premenopausal subjects experience a greater loss [37]. Even though the absolute DOSI parameters were not significantly correlated with MRI measurements at the end of NAC, the percentage change in water and TOI at the end of treatment compared with baseline did show strong correlation with the percentage change in fibroglandular breast density over the same time.

These NAC-induced changes could potentially have been caused by direct antiproliferative effects of chemotherapy, or by the indirect effect of ovarian suppression and subsequent hormone-level reductions [37]. Cytoxic chemotherapeutic agents are known to cause suppression of ovarian function and amenorrhea, whereas ovarian-secreted hormones (estrogen and progesterone) are known to increase breast density [49-51]. The greater reduction in breast-tissue water in younger, premenopausal subjects suggests that their chemo-reduced ovarian hormone levels may have a role in reducing breast-tissue density. Consequently, the change in ctO_2_Hb also suggests a hormone-independent mechanism. The enhanced breast-density reduction in premenopausal subjects could also be because breast tissue naturally becomes less dense with age [52,53]. Evidence suggests that this is also a hormonal effect [49-51]; however, it is due to natural aging and not induced by the chemotherapy. This is reflected in the reduced baseline water concentration of older subjects. If less fibroglandular tissue is present in the breasts of the older women, then it is possible that NAC may not be able to cause a significant change in breast density (that is, it has already been reduced to minimum). This complicates the ability to separate the relative importance of chemotherapy as a direct or indirect modulator of breast density.

We note that relatively little change over time was observed in mean DOSI-measured bulk lipid in both groups of patients undergoing NAC. This suggests that the rapid changes in breast density induced by NAC occur because of the reduction of the fibroglandular tissue rather than by increases or replacement by bulk lipid.

This study is limited because it is a retrospective study and includes a small number of subjects, especially those with matched DOSI and MRI measurements. Therefore, the reported correlations should be interpreted with caution, and they point to the need for further studies. Furthermore, DOSI measurements did not sample the entire breast volume. Recent data have shown, however, that density-related measurements from a spatially limited optical sampling on the breast are strongly associated with the BI-RADS category [28]. Multiple therapy regimens were included in the analysis, and it is likely that the underlying changes in breast density vary, based on the specific chemotherapy drugs given. Future work stratifying DOSI changes by treatment type and outcome in a larger population may provide insight into mechanisms of these changes, as well as patient response to therapy. Additionally, timing of the subjects' menstrual cycles was not accounted for, which can cause variations in breast density, nor their pregnancy history. Nonetheless, the ability to identify significant correlations over several years of optical measurements speaks to the strength of the DOSI method as a promising quantitative tool.

## Conclusions

In conclusion, this is the first study to confirm that optical imaging can detect significant compositional and functional changes in the contralateral normal breast before and during chemotherapy, and these changes are correlated with MRI anatomic measurements of breast density. Density is a strong independent risk factor for breast cancer, and the ability to quantify it could be valuable input to breast cancer risk models. Imaging biomarkers could be used to provide individualized treatment and predict response as well as risk of recurrence in breast cancer patients. Together with tissue analysis, DOSI may provide insight into the underlying biologic origin of density and improve our understanding of the hormonal and chemotherapy effects. Prospective studies must be performed to understand further the correlation of parameters measured with DOSI and MRI breast-imaging modalities. If validated, DOSI may provide an alternative approach to predict cancer risk as well as to monitor the protective effects of cancer therapies.

## Abbreviations

AC, doxorubicin+cyclophosphamide chemotherapy regimen; ACRIN, American College of Radiology Imaging Network; ANOVA, analysis of variance; BI-RADS, Breast Imaging-Reporting and Data System; CI, confidence interval; ct, concentration; ctHHb, tissue concentration of deoxyhemoglobin; ctO_2_Hb. tissue concentration of oxyhemoglobin; ctTHb, tissue concentration of total hemoglobin; CW-NIRS, continuous-wave near-infrared spectroscopy; DFFITS, diagnostic to assess the influence of a single point in a statistical regression; DOSI, diffuse optical spectroscopic imaging; FCMs, fuzzy-C-Means; FDPM, frequency-domain photon migration; FOV, field of view; GEE, generalized estimating equation; HER2, human epidermal growth factor receptor 2; IBIS-I, International Breast Cancer Intervention Study-I; MRI, magnetic resonance imaging; NAC, neoadjuvant chemotherapy; SERM, selective estrogen-receptor modulator; stO_2_, tissue oxygen saturation; TE, echo time (in MR imaging); TOI, tissue optical index; TR, repetition time (in MR imaging).

## Competing interests

A Cerussi and BJ Tromberg report patents owned by the University of California that are related to the technology and analysis methods described in this study. The Institutional Review Board and Conflict of Interest Office of the University of California, Irvine, have reviewed both patent and corporate disclosures and did not find any concerns. No potential conflicts of interest were disclosed by the other authors.

## Supplementary Material

Additional file 1 Title: Comparison of diffuse optical spectroscopic imaging parameters by density category
description: This table provides the mean and standard error in each diffuse optical spectroscopic imaging parameter for the pairwise comparison of Breast Imaging Reporting and Data System (BI-RADS) density classifications. Click here for file

Additional file 2 Title: Chromophore concentrations in the contralateral normal breast measured at baseline and during neoadjuvant chemotherapy
**Description: This table provides the absolute chromophore concentrations measured with diffuse optical spectroscopic imaging (DOSI) in the normal breast at baseline and at time points during neoadjuvant chemotherapy**. Click here for file
