# Supplementary material for: Optical imaging correlates with magnetic resonance imaging breast density and reveals composition changes during neoadjuvant chemotherapy
Source: Breast Cancer Res. 2013 Feb 22;15(1):R14. doi: 10.1186/bcr3389 (PMC3672664; doi:10.1186/bcr3389)
Supplement: Additional file 1 — Title: Comparison of diffuse optical spectroscopic imaging parameters by density category description: This table provides the mean and standard error in each diffuse optical spectroscopic imaging parameter for the pairwise comparison of Breast Imaging Reporting and Data System (BI-RADS) density classifications. [file bcr3389-S1.DOC]

­­­­Table S1. The mean and standard error in each diffuse optical spectroscopic imaging parameter for the pairwise comparison of Breast Imaging Reporting and Data System (BI-RADS) density classifications. CI, confidence interval; ctO_2_Hb, oxyhemoglobin concentration; ctHHb, deoxyhemoglobin concentration; ctTHb, total hemoglobin concentration; stO2, tissue oxygen saturation; TOI, tissue optical index; ScatPow, optical scattering power.

| Variable | BI-RADS II | BI-RADS III | BI-RADS IV | p-value^1,2^ | BI-RADS II vs. III | BI-RADS II vs. IV | BI-RADS III vs. IV |
| --- | --- | --- | --- | --- | --- | --- | --- |
|  | Mean (SE) | Mean (SE) | Mean (SE) |  | Mean difference  (95% CI) | Mean difference  (95% CI) | Mean difference  (95% CI) |
| Water | 18.24 (3.3) | 20.39 (1.99) | 29.45 (2.95) | 0.034^1^ | -2.15 (-10.27, 5.98) | -11.21 (-20.54, -1.87)* | -9.06 (-16.57, -1.55) |
| Lipid | 75.87 (3.37) | 70.01 (2.03) | 63.98 (3.01) | 0.054^1^ | 5.86 (-2.44, 14.16) | 11.89 (2.36, 21.43) | 6.03 (-1.64, 13.7) |
| ctO_2_Hb | 18.6 (2.92) | 14.75 (1.76) | 23.24 (2.61) | 0.047^1^ | 3.85 (-3.35, 11.05) | -4.64 (-12.91, 3.63) | -8.49 (-15.14, -1.84)* |
| ctHHb | 4.89 (0.46) | 4.69 (0.27) | 6.23 (0.41) | 0.019^1^ | 0.2 (-0.93, 1.32) | -1.34 (-2.63, -0.05) | -1.54 (-2.57, -0.5)* |
| ctTHb | 23.49 (3.06) | 19.44 (1.85) | 29.47 (2.74) | 0.025^1^ | 4.04 (-3.5, 11.59) | -5.98 (-14.65, 2.68) | -10.02 (-16.99, -3.06)* |
| stO_2_ | 77.97 (2.68) | 75.32 (1.61) | 77.43 (2.39) | 0.636^2^ | 2.64 (-3.95, 9.24) | 0.54 (-7.04, 8.11) | -2.11 (-8.2, 3.99) |
| TOI | 1.17 (0.5) | 1.47 (0.3) | 3.14 (0.45) | 0.028^2^ | -0.3 (-1.53, 0.93) | -1.97 (-3.38, -0.56)* | -1.67 (-2.8, -0.53)* |
| ScatPow | -0.79 (0.11) | -0.64 (0.07) | -0.73 (0.1) | 0.483^1^ | -0.15 (-0.42, 0.13) | -0.06 (-0.37, 0.26) | 0.09 (-0.16, 0.35) |

^1^p-value of type III test from analysis of variation (ANOVA) analysis.

^2^p-value of Kruskal-Wallis test.

*p-value < 0.05 for the comparison after the Bonferroni-Holm method of adjustment for multiple comparisons was applied.
