# Supplementary material for: Optical imaging correlates with magnetic resonance imaging breast density and reveals composition changes during neoadjuvant chemotherapy
Source: Breast Cancer Res. 2013 Feb 22;15(1):R14. doi: 10.1186/bcr3389 (PMC3672664; doi:10.1186/bcr3389)
Supplement: Additional file 2 — Title: Chromophore concentrations in the contralateral normal breast measured at baseline and during neoadjuvant chemotherapy Description: This table provides the absolute chromophore concentrations measured with diffuse optical spectroscopic imaging (DOSI) in the normal breast at baseline and at time points during neoadjuvant chemotherapy. [file bcr3389-S2.DOC]

Table S1: Absolute measured chromophore concentrations in the normal breast measured at baseline and during NAC (mean ± standard error). ctO_2_Hb, oxyhemoglobin concentration; ctHHb, deoxyhemoglobin concentration; ctTHb, total hemoglobin concentration; stO_2_, tissue oxygen saturation; TOI, tissue optical index

|  | **Baseline** | **~30 days** | **~60 days** | **~90 days** | **~120 days** |
| --- | --- | --- | --- | --- | --- |
| **Water (%)** |  | | | | |
| All | 21.4 ± 1.3 | 20.6 ± 1.1 | 19.1 ± 1.0 | 19.6 ± 1.0 | 19.1 ± 1.1 |
| Pre | 24.4 ± 1.8*** | 23.2 ± 1.6** | 20.8 ± 1.3* | 20.9 ± 1.4 | 20.2 ± 1.6 |
| Post | 16.6 ± 0.7*** | 16.9 ± 0.8** | 16.6 ± 0.8* | 17.2 ± 0.8 | 17.4 ± 1.0 |
| **Lipid (%)** |  | | | | |
| All | 69.7 ± 1.3 | 69.8 ± 1.3 | 70.1 ± 1.1 | 70.5 ± 1.1 | 70.0 ± 1.3 |
| Pre | 67.0 ± 1.6** | 67.4 ± 1.7* | 69.0 ± 1.4 | 68.9 ± 1.3 | 69.6 ± 1.6 |
| Post | 74.0 ± 1.4** | 73.3 ± 1.5* | 71.7 ± 1.8 | 73.4 ± 1.7 | 70.6 ± 2.5 |
| **ctO_2_Hb (µM)** |  | | | | |
| All | 18.0 ± 1.2 | 16.3 ± 1.1 | 10.9 ± 1.0 | 13.9 ± 1.3 | 12.2 ± 1.1 |
| Pre | 18.9 ± 1.7 | 17.1 ± 1.2 | 12.6 ± 1.2 | 14.7 ± 1.7 | 12.6 ± 1.1 |
| Post | 16.6 ± 1.5 | 15.1 ± 2.0 | 8.5 ± 1.4 | 12.6 ± 1.7 | 11.5 ± 2.2 |
| **ctHHb (µM)** |  | | | | |
| All | 5.0 ± 0.2 | 4.9 ± 0.2 | 5.0 ± 0.3 | 4.9 ± 0.2 | 4.8 ± 0.4 |
| Pre | 5.3 ± 0.2* | 5.3 ± 0.3 | 5.4 ± 0.3 | 5.1 ± 0.3 | 5.2 ± 0.6 |
| Post | 4.5 ± 0.2* | 4.4 ± 0.3 | 4.5 ± 0.4 | 4.5 ± 0.3 | 4.2 ± 0.3 |
| **ctTHb (µM)** |  | | | | |
| All | 23.0 ± 1.3 | 21.2 ± 1.2 | 15.9 ± 1.0 | 18.9 ± 1.3 | 17.0 ± 1.2 |
| Pre | 24.2 ± 1.9 | 22.3 ± 1.4 | 17.9 ± 1.2* | 19.8 ± 1.8 | 17.8 ± 1.5 |
| Post | 21.1 ± 1.6 | 19.5 ± 2.1 | 13.0 ± 1.2* | 17.2 ± 1.8 | 15.7 ± 2.2 |
| **stO_2_ (%)** |  | | | | |
| All | 77.2 ± 0.9 | 75.7 ± 1.1 | 66.3 ± 2.9 | 71.4 ± 1.9 | 71.0 ± 2.1 |
| Pre | 76.8 ± 1.1 | 75.7 ± 1.2 | 69.0 ± 2.1 | 71.7 ± 2.1 | 70.8 ± 2.1 |
| Post | 77.7 ± 1.7 | 75.7 ± 2.3 | 62.4 ± 6.5 | 71.0 ± 4.1 | 71.3 ± 4.9 |
| **TOI** |  | | | | |
| All | 1.7 ± 0.2 | 1.6 ± 0.2 | 1.4 ± 0.1 | 1.5 ± 0.1 | 1.4 ± 0.2 |
| Pre | 2.1 ± 0.3** | 2.0 ± 0.3** | 1.7 ± 0.2* | 1.7 ± 0.2 | 1.5 ± 0.2 |
| Post | 1.0 ± 0.1** | 1.0 ± 0.1** | 1.1 ± 0.1* | 1.1 ± 0.1 | 1.1 ± 0.2 |

*, **, *** denote a statistically significant difference (Mann-Whitney U test) between pre- and post-menopausal groups (p<0.05, p<0.01,p<0.001)
